# Clarification of the molecular pathway of Taiwan local pomegranate fruit juice underlying the inhibition of urinary bladder urothelial carcinoma cell by proteomics strategy

**DOI:** 10.1186/s12906-016-1071-7

**Published:** 2016-03-09

**Authors:** Ting-Feng Wu, Li-Ting Hsu, Bo-Xian Tsang, Li-Chien Huang, Wan-Yin Shih, Li-Yi Chen

**Affiliations:** Department of Biotechnology Southern Taiwan University of Science and Technology, 1 Nan-Tai Street, YungKang District, 710, Tainan, Taiwan

**Keywords:** Apoptosis, Urinary bladder urothelial carcinoma, Pomegranate, Proteomics, Akt

## Abstract

**Background:**

Pomegranate fruit has been shown to exhibit the inhibitory activity against prostate cancer and lung cancer in vitro and in vivo, which might be a resource for chemoprevention and chemotherapy of cancer. Our previous documented findings indicated that treatment of urinary bladder urothelial carcinoma cell with the ethanol extract isolated from the juice of pomegranate fruit grown in Taiwan could inhibit tumor cell. In this study we intended to uncover the molecular pathway underlying anti-cancer efficacy of Taiwan pomegranate fruit juice against urinary bladder urothelial carcinoma.

**Methods:**

We exploited two-dimensional gel electrophoresis coupled with tandem mass spectrometry to find the de-regulated proteins. Western immunoblotting was used to confirm the results collected from proteomics study.

**Results:**

Comparative proteomics indicated that 20 proteins were differentially expressed in ethanol extract-treated T24 cells with 19 up-regulated and 1 down-regulated proteins. These de-regulated proteins were involved in apoptosis, cytoskeleton regulation, cell proliferation, proteasome activity and aerobic glycolysis. Further studies on signaling pathway demonstrated that ethanol extract treatment might inhibit urinary bladder urothelial carcinoma cell proliferation through restriction of PTEN/AKT/mTORC1 pathway via profilin 1 up-regulation. It also might evoke cell apoptosis through Diablo over-expression.

**Conclusions:**

The results of this study provide a global picture to further investigate the anticancer molecular mechanism of pomegranate fruit.

**Electronic supplementary material:**

The online version of this article (doi:10.1186/s12906-016-1071-7) contains supplementary material, which is available to authorized users.

## Background

Bladder cancer is the most prevalent tumor of the urinary tract worldwide and ranked the 15^th^ in cancer mortality rate in 2011 in Taiwan [1, 2]. Urothelial carcinoma is the most common and constitutes more than 90 % of bladder cancer cases in developed countries [3]. According to WHO/ISUP classification (2004), urinary bladder urothelial carcinoma (UBUC) cell can be classified into low and high grade while high grade UBUC cell is less differentiated. Although most of UBUCs are papillary/non-invasive or superficially invasive types and can often be cured by curettage, some UBUCs can still develop relentless local recurrence followed by lethal distal spreading [4].

Pomegranate (*Punicagranatum*, Punicaceae), is an edible fruit cultivated in Mediterranean countries, India and the United States etc., comprising edible portions of 80 % juice and 20 % seeds. Pomegranate contains crude fibers, pectin, sugars, tannins (mainly ellagitannins), flavonoids and anthocyanins. Among those nutritious ingredients, anthocyanins is believed to have provided the fruit with potent antioxidant ability [5].

Many literatures have showed that pomegranate fruit displays anti-cancer effectiveness. Ellagitannin-rich pomegranate fruit extract (PFE) purified from pomegranate edible portion with 70 % acetone was found to have the apoptotic effects on human lung cancer A549 cells through the down-regulation of cell cycle-regulatory proteins operative in the G1 stage and inhibiting NF-κB as well as MAP kinase pathways [p38, phosphoinositide 3 kinase (PI3K)/ protein kinase B (Akt), c-jun N-terminal kinase (JNK) and extracellular signal-regulated kinase (Erk)] [6]. In the animal model of primary lung tumor, PFE also diminishes tumor growth/progression/angiogenesis by the suppression of NF-κB, MAP kinase pathways and mammalian target of rapamycin (mTOR) signaling [7]. In addition to the impacts on lung cancer, PFE may be a potential source for chemoprevention of prostate cancer [8]. The investigations on prostate cancer found that pomegranate polyphenols, ellagitannin-rich extract (PE) prepared from fruit skins can retard prostate cancer likely caused by chronic inflammation via suppressing the NF-κB pathway [9]. PE was also observed to down-regulate the angiogenesis in prostate cancer through down-regulation of hypoxia-inducible factor 1-α (HIF-1α) which transcriptionally regulates the vascular endothelial growth factor (VEGF) expression [10]. Clinical trial with rising prostate-specific antigen (PSA) after surgery or radiotherapy indicated that after daily treatment with 8 oz of pomegranate juice a significant increase in mean PSA doubling time from 15 months at baseline to 54 months post-treatment [11]. Our previous documented results showed that pomegranate juice could evoke prostate cancer cell apoptosis via mitochondrial pathway and death receptor signaling. It also can interfere with the expression levels of genes involved in cytoskeletal functions, anti-apoptosis, metabolism, NF-B signaling in juice-treated prostate cancer cell [12].

Based upon the aforementioned documented findings, pomegranate may be a potential chemopreventive resource against UBUC development and recurrence.

Our previous documented findings indicated that treatment of the ethanol extract (PEE) of pomegranate fruit juice could inhibit UBUC cell via cell cycle arrest and cell apoptosis [13]. Thus in this study we exploited two-dimensional gel electrophoresis (2-DE) coupled with tandem mass spectrometry to decipher the molecular mechanism underlying the cancer intervention of PEE. We found that PEE treatment could inhibit UBUC cell proliferation and migration. PEE-induced de-regulated proteins were associated with apoptosis, cytoskeleton regulation, AKT/mTOR signaling, proteasome activity and aerobic glycolysis. These de-regulated proteins might contribute to PEE-evoked inhibition of UBUC cell proliferation and cell apoptosis.

## Methods

### Collection and identification of plant materials

The fruits of *P. granatum* were field collected from a farm land (22°41′59.3267″ N, 120°30′45.1836″ E) located in a small township Jiuru, Pingtung county, southern Taiwan from August to September, 2012. The plant specimens were identified by Dr. Gwo-Ing Liao from National Chen-Kung University, Taiwan and were pressed/dried for voucher specimens (Nan-Kai Lin, STUSTG308-001 to STUSTG308-003) deposited in the herbarium of Taiwan forestry research Institute (TAIF), Taiwan.

### Preparation of ethanol extract (PEE) of pomegranate fruit juice

PEE was prepared as described previously [13]. In brief, fresh pomegranate fruit was peeled and juice was concentrated by freeze drying. The powder was first extracted with ethylacetate (EtOAc) at a ratio of 1:3 (w/v). After extraction, the residue was collected by centrifugation and then extracted with 70 % (v/v) ethanol as described in EtOAc extraction. The supernatant of ethanol extraction was vaccum dried and the product was recognized as PEE which was stored at −20 °C till future use. Appropriate amount of PEE dissolved in DMSO was used for anti-cancer assay and proteomics study.

### Cell lines

Human UBUC T24 and J82 cells were used in this investigation. Human UBUC T24 cell, which is recognized as high grade and invasive, was purchased from Bioresource Collection and Research Center, Hsinchu, Taiwan and cultured at 37 °C in McCoy’s5A [GIBCO (Life technologies), Grand Island, N.Y., U.S.A.], supplemented with 10 % (v/v) fetal bovine serum (FBS). UBUC J82 cells recognized as high grade was offered by Dr. Chien-Feng Li from Department of Pathology, Chi-Mei Medical Center, Tainan, Taiwan and maintained at 37 °C in Dulbecco’s Modified Eagle Medium supplemented with 10 % (v/v) FBS (GIBCO, Grand Island, N.Y., U.S.A.). TSGH8301 cell (low grade) was derived from patients with superficial bladder cancer in Taiwan [14] and provided by Dr. Chien-Feng Li in 2010 from Department of Pathology, Chi-Mei Medical Center. TSGH8301 cell was cultivated at 37 °C in RPMI-1640 (GIBCO)/10 % (v/v) FBS.

### Isoelectric focusing (IEF) and SDS-polyacrylamide gel electrophoresis (SDS-PAGE)

Preparation of protein lysates for two-dimensional gel electrophoresis was described in an additional file [Additional file 1]. IEF and SDS-PAGE was undertaken as described before with some modifications [12]. The pH 4–7, 18-cm immobibline dry strips (GE Healthcare Bio-Sciences AB, Uppsala, Sweden) were rehydrated using BioRad Protean IEF Cell for 16 h at 20 °C with 300 μl rehydration buffer containing 100 μg protein lysates prepared from PEE-exposed or 0.5 % (v/v) DMSO (vehicle)-exposed T24 cells. The proteins were then focused at 20 °C at 50 V, 100 V, 200 V, 500 V, 1000 V, 5000 V and 8000 V respectively with a total of 81,434 voltage-hours.

### Image analysis and statistical analysis

2-DE gels were stained with LavaPurple™ according to manufacture’s protocol described in brief in an additional file [Additional file 1]. Then the images of 2-DE gel map were scanned using Typhoon 9400 fluorescence scanner (GE healthcare) with green laser (green laser PMT: 600 volt and emission filter: 580 BP). To search for the de-regulated proteins in PEE-exposed T24 cells for 36 h, a total of 9 pairs of well-focused gel maps collected from control and PEE-treated T24 cells were compared by PDQuest 8.0.1 (BioRad) software. Dys-regulated expressed protein spots identified by computer analysis were further confirmed by visualization. The intensity of the spot was measured and normalized as a percentage of the total intensities of all spots in a gel (total normalized volume). For each differentially expressed protein spot normalized volumes of individual protein spots across replica gels of 0.5 % (v/v) DMSO- or PEE-incubated T24 cells were first analyzed by the normal distribution test and then Student’s *t*-Test (STATISTICA, StatSoft, Tulsa, OK, U.S.A.) was carried out when normal distribution was obtained. However, when normal distribution was not acquired, log transformation was performed followed by the normal distribution test and Student’s *t*-Test. In all cases, statistical variance of the PEE-exposed : control spot intensity ratio within 95 % (Student’s *t*-Test; *P* < 0.05) was considered to be significantly different. Furthermore the differentially expressed proteins present at least in 5 out of 9 gel pairs were regarded as PEE-impacted proteins.

### In-gel digestion and protein identification analysis via liquid chromatography-tandem mass spectrometry (LC-MS/MS)

The protein spots of interest were picked up for in-gel digestion using silver staining. The silver staining, in-gel digestion and mass spectrometric protein identification were performed as described previously [12]. Briefly, the protein digest was separated in LTQ-Orbitrap hybrid tandem mass spectrometer (ThermoFisher, USA) in-line coupled with Agilent 1200 nanoflow HPLC system equipped with LC Packing C18 PepMap 100 (length: 5 mm; internal diameter: 300 μm; bead size: 5 μm) as the trap column and Agilent ZORBAX XDB-C18 (length: 50 mm; internal diameter: 75 μm; bead size: 3.5 μm) as the separating column. File Converter in Xcalibur 2.0SR package (ThermoFisher, USA) and an in-house program were used to extract the MS/MS information as well as to compute the charge and mass for each analyzed peptide. TurboSequest program (ver. 27, rev. 11) was then used to search the best matched peptides from a non-redundant protein database whose FASTA sequences were downloaded from National Center for Biotechnology Information (http://www.ncbi.nlm.nih.gov/guide/proteins/#tab-all_) on 2010/10/12 with 541927 entries. While only the tryptic peptides with ≦ 2 missed cuts were considered, the mass ranges during the database search were 1 and 3.5 *m/z* for fragment and precursor ions respectively. The protein identities were verified only when there were at least two peptides matched and both search results had high Xcore (i.e., ≧ 2.0 for doubly charged peptides and ≧3.0 for triply charged ones) and with minimal differences between observed and hypothetical masses (i.e., ΔM <10 ppm). For each set of MS/MS analyses, 25 fmol of BSA in gel was analyzed in parallel for verification of effectiveness of the entire protein identification procedure, including in-gel digestion, nanoflow HPLC, MS/MS and informatics analyses. The experimental data were only taken into account only when 10 ppm mass accuracy and over 70 % coverage was observed for the co-processed BSA sample.

### Western immunoblotting

After treatment as described in section 3, T24 or J82 cells were harvested and lysed in lysis buffer [10 mMTris (pH 8.0), 0.32 M sucrose, 1 % (v/v) Triton X-100, 5 mM EDTA, 2 mM DTT, and 1 mM PMSF]. After determining its protein concentration using Bio-Rad DC protein assay kit, equal volume of 2× sample buffer [0.1 M Tris (pH 6.8), 2 % (w/v) SDS, 0.2 % (v/v) β-mercaptoethanol, 10 % (v/v) glycerol, and 0.0016 % (w/v) bromophenol blue] was combined with the protein lysate. Appropriate amounts of the lysates were separated by electrophoresis at 100 V with 10 % (w/v) SDS-PAGE, and further transferred onto a PVDF membrane (Strategene, La Jolla, CA, USA). After blocking for 1 h in 3 % (w/v) bovine albumin serum (BSA) at room temperature, membranes were hybridized overnight at room temperature with primary antibodies listed in an additional file [Additional file 1]. The membranes were washed and probed with suitable secondary antibodies for 1 h at room temperature. Secondary antibodies binding on the membrane were detected by chemiluminescence ECL detection system (GE Healthcare Bio-Sciences AB, Uppsala, Sweden) using Fujifilm LAS-3000 Luminescent Image Analyzer (Fujifilm Corporation, Tokyo, Japan). The intensity of each protein band, normalized with actin or glyceraldehyde-3-phosphate dehydrogenase (GAPDH) protein expression level, was quantified by PDQUEST Quantity One software (Bio-Rad Laboratory, Hercules, CA) and analyzed with Student’s *t*-Test (STATISTICA, StatSoft, Tulsa, OK, U.S.A.).

## Results

### Inhibitory impacts of PEE on outgrowth and migration of UBUC cell

**Fig. 1:**
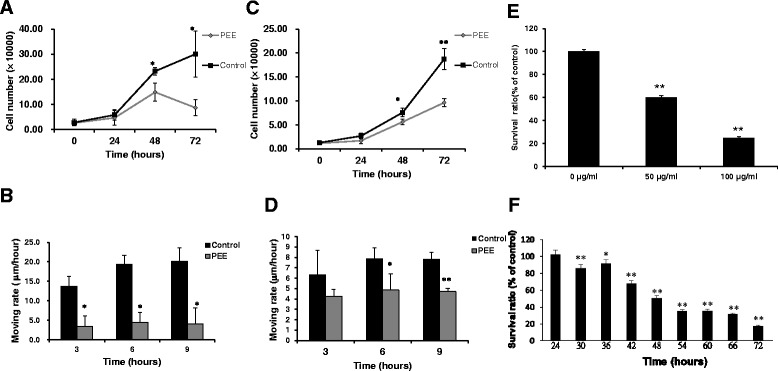
Inhibitory influences of PEE on UBUC cells. **a** T24 cell proliferation assay. T24 cells were incubated with 50 μg/mL PEE for the indicated times and cell proliferation was measured by cell counting assay as described in an additional file [Additional file 1]. The diagram was the typical result of three independent experiments. All the data were expressed as mean ± standard deviation (S.D.) of the mean of four wells. **b** T24 cell migration. T24 cells were incubated with 50 μg/mL of PEE for the indicated times and cell migration was observed as described in an additional file [Additional file 1]. The results were expressed as mean ± S.D. of the mean of three independent experiments. **c** J82 cell proliferation assay. **d** J82 cell migration. **e** TSGH8301 cell survival assay. **f** T24 cell survival assay for proteomics study. T24 cells were treated with 50 μg/mL PEE for the indicated durations and cell viability was measured by MTT assay as described in an additional file [Additional file 1]. 0.5 % (v/v) DMSO-treated cells were recognized as the control in all the assays. TSGH8301 cell was incubated with the indicated doses of PEE for 72 h and cell viability was measured by MTT assay. 0 μg/ml indicated DMSO control. * and ** represented *P* < 0.05 and *P* < 0.001 respectively as compared to untreated cell using Student’s *t*-test

Our previous documented data demonstrated that PEE might initiate a cascade of biochemical events to induce UBUC cell apoptosis. In this study, we found that PEE treatment temporally retarded T24 cell proliferation and also could slow down T24 cell migration. The comparable impacts were also observed in PEE-incubated J82 cells (Fig. 1). PEE incubation also exerted the inhibitory effects on low grade UBUC TSGH8301cell proliferation (Fig. 1).

### Two-dimensional gel electrophoresis of PEE-treated T24 cells

To uncover the molecular mechanism underlying the effects evoked by PEE, Lavapurple stained 2-DE gels coupled with LC-MS/MS were conducted to profile protein expression and search for those proteins whose expressions were altered by PEE exposure. Since PEE treatment showed a better impact on T24 cell proliferation /migration, T24 cell was chosen for subsequent proteomics study. T24 cells treated for 36 h with or without 50 μg/mL PEE were selected for 2-DE analyses because, as indicated by the results shown by Fig. 1f, sufficiently affected cells that were at the same time still viable could be collected and potentially relevant cellular changes prior to cell death could typically be observed in PEE-exposed T24 cells.

**Fig. 2:**
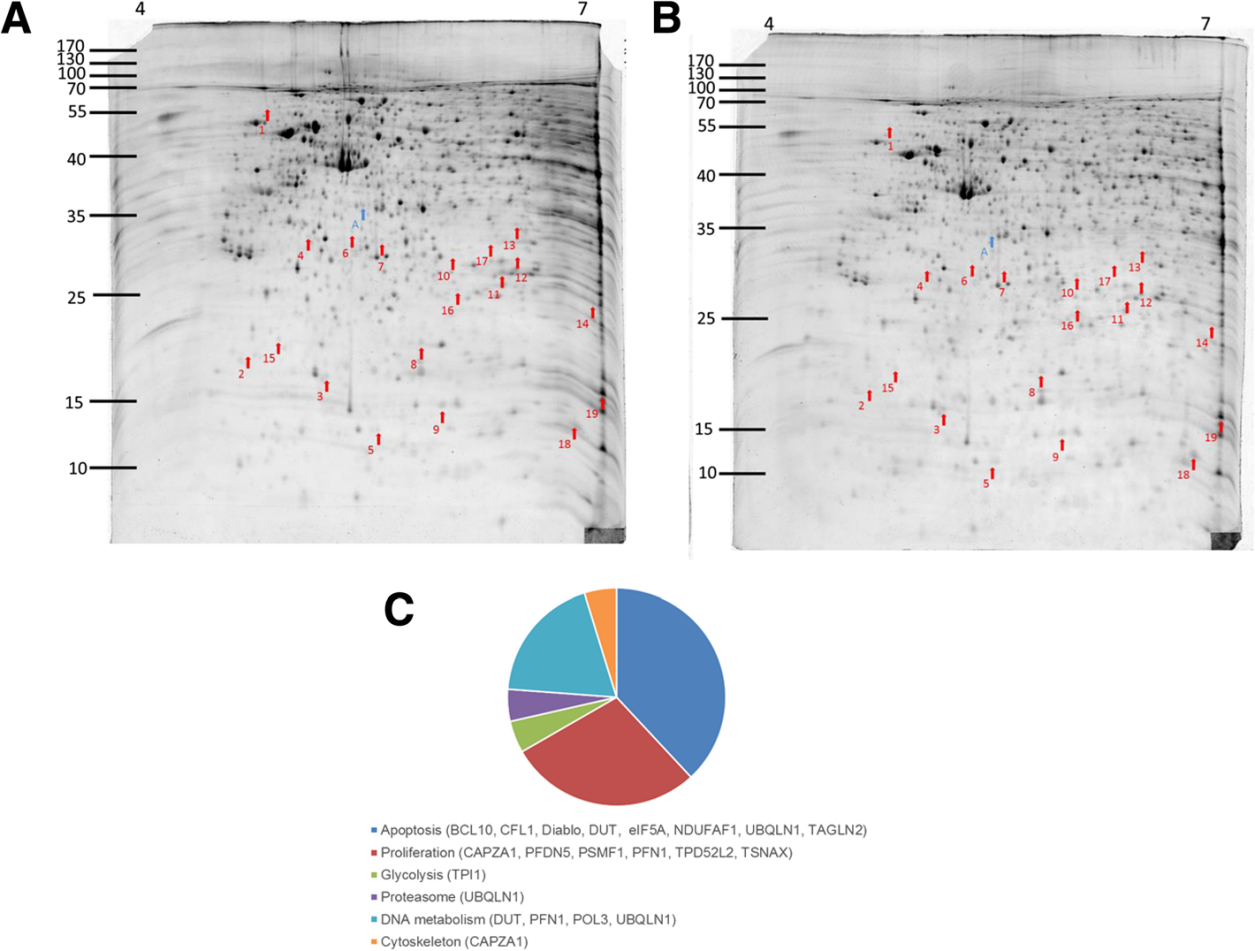
Lavapruple stained 2-DE polyacrylamide gel maps of untreated and PEE-treated T24 cells. The gel pair shown was the representative of 9 pairs of (**a**) 0.5 % (v/v) DMSO-incubated T24 cells and (**b**) PEE-incubated T24 cells. Protein lysates were prepared from 50 μg/mL PEE-treated human T24 cells for 36 h as mentioned in the supplementary data. 100 μg proteins were loaded on linear pH 4–7 gradient strip followed by vertical separation on 12.5 % (w/v) SDS polyacrylamide gels as described in section 2. The numbers indicated on the top of the gel indicated the pH range and those on the left shown the approximate molecular mass (kDa) determined by Bio-Rad protein markers. The differentially expressed spots were demonstrated by arrows. 19 spots were found to be up-regulated (spots 1–19) in PEE-affected cell and 1 spot was under-expressed (spot A). **c** IPA Results

Initially 100 μg of proteins from mock and PEE-incubated T24 cells were loaded and separated by 2-DE of 18-cm gel strips (pI 4–7). To prevent the gel-to-gel variation, nine replicate gel pairs were collected from three independent treatments. The representative 2-DE maps of un-treated and PEE-treated T24 cells were depicted in Fig. 2 and the remaining 8 gel pairs were shown in an additional file [Additional file 1].

The proteome maps of control and PEE-exposed T24 cells were compared to recognize the protein spot variations. After PEE treatment, differentially expressed protein features were recorded as described in Methods. Nineteen up-regulated and one down-regulated protein spots were found as denoted by the arrowed spots in Fig. 2. Spots 3, 18 and A were close to 2-fold but statistical significant (*P* < 0.05). The total normalized volume (ppm) and statistical result of each protein spot were presented in [Additional file 2: Table S1].

### Identification of the differentially expressed proteins in PEE-influenced T24 cells

After the proteome comparison, de-regulated proteins were identified using LC-MS/MS as described in Methods. The results of spectrometric analyses were summarized in Table 1 and the matched peptides of each de-regulated protein were shown in [Additional file 2: Table S2]. The experimental molecular weight and pI of each protein spot were near the theoretical values, and most of the spectrometric protein coverages were above 20 %.

**Table 1:** Differentially expressed proteins identified by tandem mass spectrometry^a^

Spot	Protein identity	Incidences	Experiment pI/MW (kDa)	Theoretical pI/MW (kDa)	Matched^b^ peptide number	Coverage (%)	Accession number (NCBI)	Fold
1	ubiquilin 1 (UBQLN1)	5/9	4.8/56.2	5.2/62.5	10	10.34	119583063	+2.73
2	DNA polymerase ε subunit 3 (POLE3)	5/9	4.7/20.2	4.68/16.86	6	32.65	119607787	+7.58
3	eukaryotic translation initiation factor 5A (eIF5A)	6/9	5/19.6	5.07/16.83	4	36.36	119610625	+1.78
4	tumor protein D54 (TPD52L2)	6/9	4.9/29.9	5.26/22.24	8	44.66	11125673	+12.84
5	phosphohistidine phosphatase 1 (PHPT1)	6/9	5.3/17.6	5.65/13.83	2	22.4	11191302	+5.12
6	proteasome inhibitor subunit 1 (PSMF1/PI31)	5/9	5.1/32.3	5.42/29.82	6	25.83	10432572	+3.97
7	B-cell lymphoma/leukemia 10 (BCL 10)	5/9	5.3/31.8	5.57/26.25	3	12.02	119593614	+2.9
8	dUTP pyrophosphatase (DUT)	5/9	5.5/21.2	6.15/17.75	5	42.07	10257385	+6.81
9	prefoldin subunit 5 (PFDN5)	5/9	5.8/19	5.94/17.33	9	57.79	119617089	+2.51
10	peflin (PEF1)	7/9	5.8/30.8	4.84/28.22	7	23.6	119628003	+6.31
11	triosephosphate isomerase 1 (TPI1)	5/9	6.2/28	5.65/30.79	8	39.86	119609128	+6.27
12	26S proteasome non-ATPase regulatory subunit 9 (PSMD9)	5/9	6.3/30.5	6.46/24.68	2	9.42	119618700	+7.17
13	NADH dehydrogenase (ubiquinone) 1 α subcomplex assembly factor 1 (NDUFAF1)	5/9	6.3/34.9	7.11/37.76	4	17.43	119612893	+5.2
14	transgelin 2 (TAGLN2)	5/9	6.9/22.6	8.41/22.39	10	53.27	119573145	+3.2
15	Diablo (DIABLO)	5/9	4.8/21.5	5.67/27.16	10	43.51	10437144	+3.94
16	BCL2-associated athanogene 2 (BAG2)	5/9	5.9/26.3	6.25/23.77	9	43.60	115528700	+4.71
17	translin-associated factor X (TSNAX)	5/9	6.2/33.3	6.10/33.11	8	42.41	119590369	+6.54
18	profilin 1 (PFN1)	5/9	6.8/17.9	8.44/15.05	7	65.00	119610787	+1.57
19	cofilin (CFL1)	5/9	7.0/19.0	8.22/18.50	10	56.02	116848	+2.86
A	F-actin-capping protein subunit alpha-1 (CAPZA1)	5/9	5.2/36.8	5.45/32.92	10	41.26	119576929	−1.93

^a^Matched peptide number: Number of peptides matched with protein in MS/MS query. Coverage: Total percentage of amino acid sequence covered by peptides identified by MS/MS analyses

^b^The detail data of MS/MS identification for each peptide was provided in Additional file 2: Table S2, supporting information

The over-expressed proteins (spots 1–19) and under-expressed proteins (spot A) were identified as described in Table 1. Results of Ingenuity pathway analysis (IPA) (Qiagen) showed that the identified de-regulated proteins were involved in cell apoptosis (BCL10, CFL1, Diablo, DUT, eIF5A, NDUFAF1, UBQLN1, TAGLN2), cell proliferation (CAPZA1, PFDN5, PSMF1, PFN1, TPD52L2, TSNAX), DNA metabolism (DUT, PFN1, POL3, UBQLN1), proteasome (UBQLN1), glycolysis (TPI1), cytoskeleton (CAPZA1) (Fig. 2c).

### Confirmation of differentially expressed proteins

**Fig. 3:**
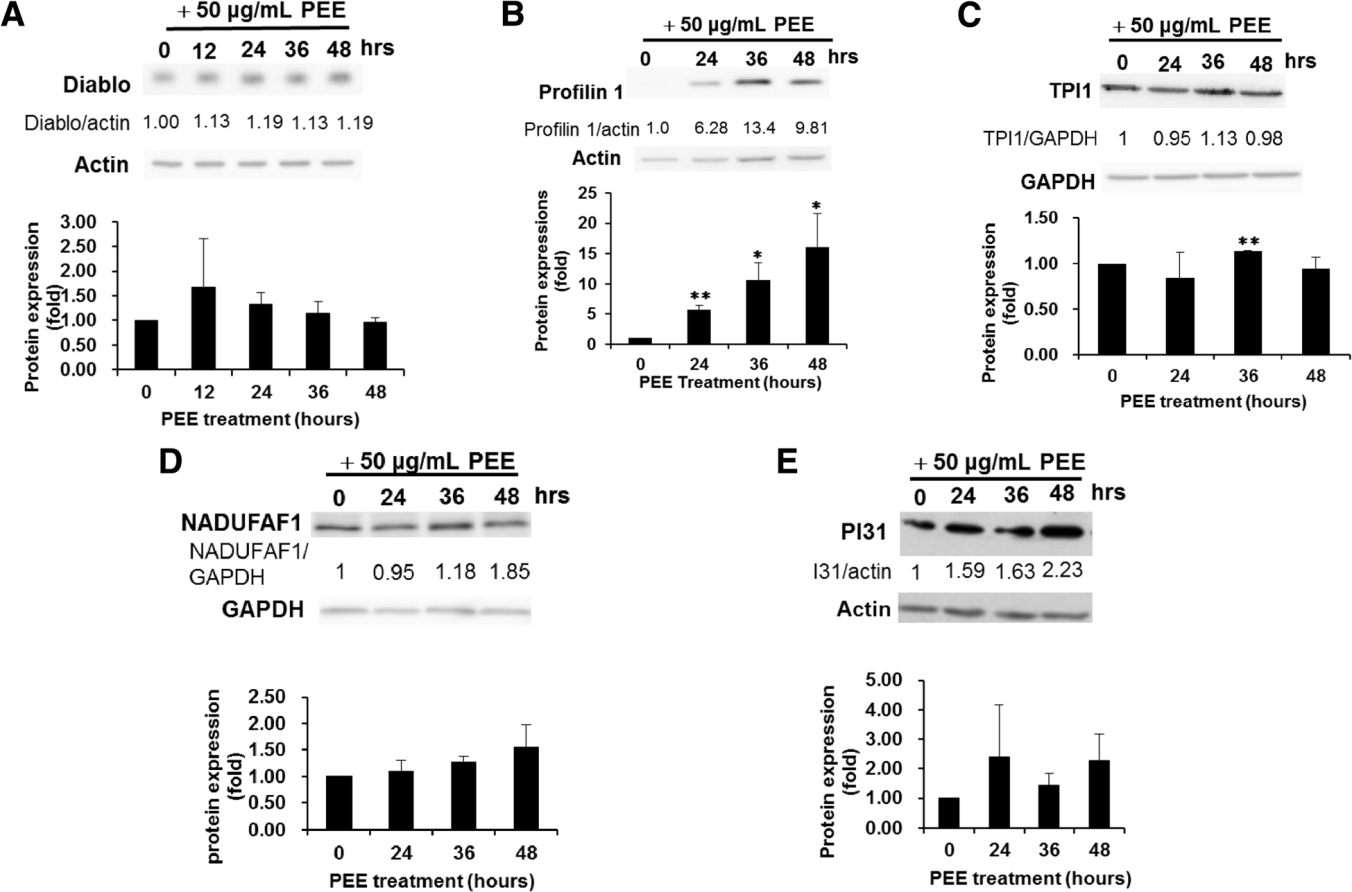
Confirmation of the impacts of PEE on the expression levels of diablo, profilin 1, TPI1, NADUFAF1, PI31 in T24 cell by western immunoblotting. **a** Diablo. **b** Profilin 1. **c** TPI1. **d** NADUFAF1. **e** PI31. Human T24 cells were incubated with 50 μg/mL PEE for various durations (hours). Western immunoblotting was carried out as described in section 2. The blot was the typical data of three independent studies. The protein expression fold (mean ± S.D.) was expressed as the ratio of normalized intensity (observed protein/actin or GAPDH) at each time point divided by that at the beginning of treatment (0 h). * and ** represented *P* < 0.05 and *P* < 0.001 respectively as compared to untreated cell using Student’s *t*-test

To validate our observation on the proteomics results, western immunoblotting was exploited to evaluate the expressions of Diablo, profilin 1, TPI1, NDUFAF1 and PSMF1/PI31. These five proteins were targeted based on their respective high-fold alterations except profilin 1 and potential relevance to cell proliferation and apoptosis. Consistent with the proteomics data, the results of western immunoblotting demonstrated that Diablo, profilin 1, TPI1, NDUFAF1 and PSMF1/PI31 were up-regulated in PEE-treated T24 cells for 36 h (Fig. 3).

**Fig. 4:**
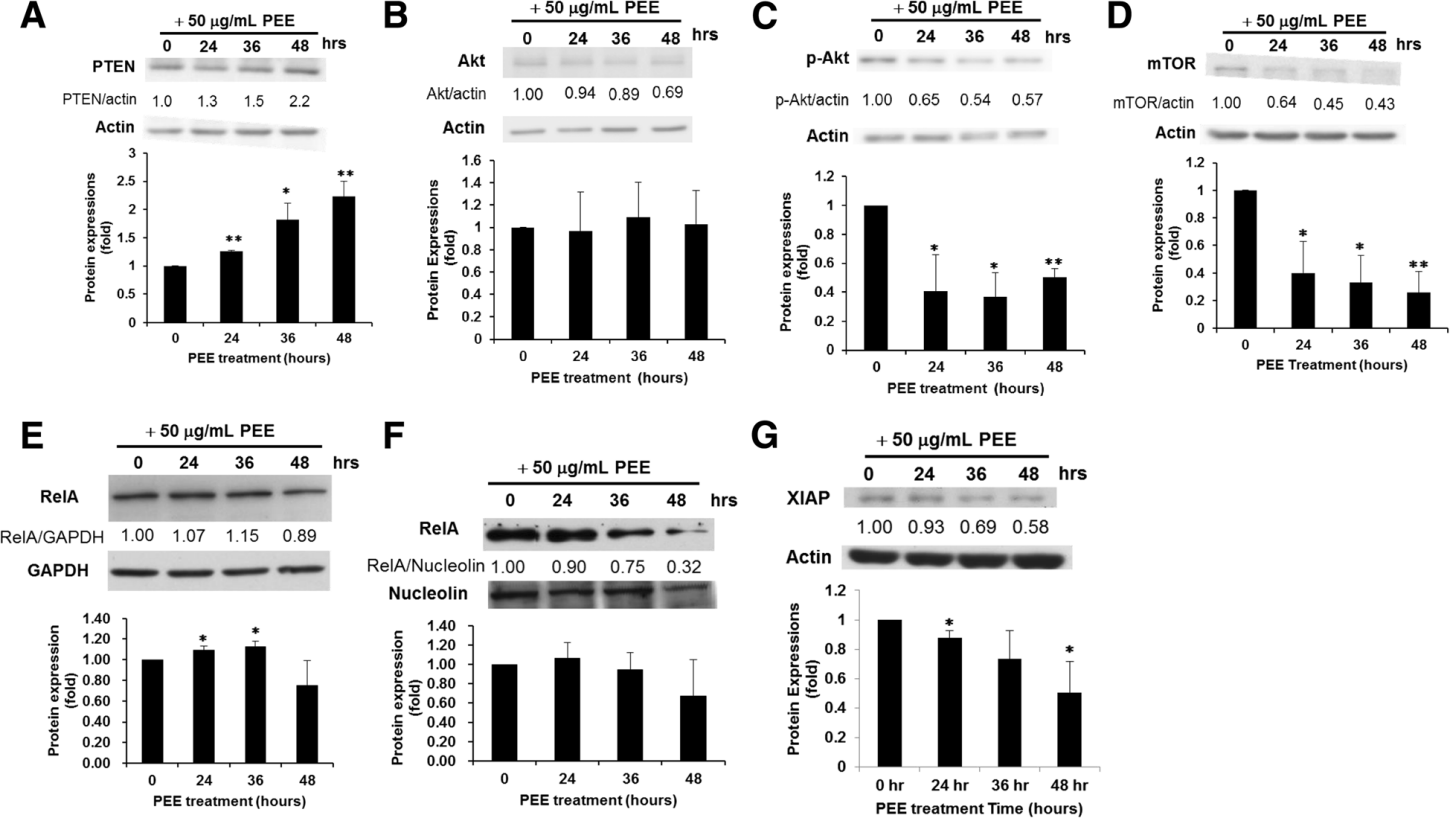
Effects of PEE on the expression levels of PTEN/Akt/mTOR pathway, NFκB and XIAP in T24 cell. **a** PTEN. **b** Akt. **c** p-Akt. **d** mTOR. **e** Rel A in cytosol. **f** Rel A in nucleus. **g** XIAP. An additional file [Additional file 1] showed the preparation of nuclear and cytoplasmic extracts. Legends were the same as those in Fig. 3

**Fig. 5:**
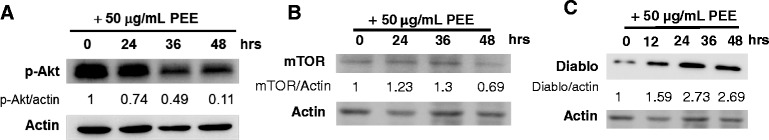
Impacts of PEE on the expression levels of Diablo, p-Akt and mTOR in TSGH8301 cell. **a** p-Akt. **b** mTOR. **c** Diablo

Previous findings demonstrated that profilin 1 over-expression increases phosphatase and tensin homolog (PTEN) protein synthesis while PTEN gene up-regulation inhibits Akt activity [15, 16]. In accordance with previous findings our study showed that the exposure of T24 cell to 50 μg/mL PEE could raise PTEN gene expression probably due to profilin 1 over-expression and in turn dwindled phospho-Akt expression, which barely influenced Akt protein expression (Fig. 4). Furthermore, PEE treatment could inhibit the expression of mTOR phosphorylated at S2448, which is the catalytic subunit of mTORC1 complex [17] (Fig. 4) and thus might interfere with the nutrient metabolism or energy production in cancer cell. It has been suggested that mTORC1 contains mTOR phosphorylated predominantly on S2448 and inhibition of mTOR S2448 phosphorylation correlates with the decreased mTORC1 activity [17]. Reduced expression levels of p-Akt and downstream phosphorylated mTOR (S2448) were also observed in PEE-treated TSGH8301 cell (Fig. 5). The above results suggested that through the up-regulation of profilin 1 expression PEE exposure could inhibit Akt/mTOR pathway which plays a significant role in carcinogenesis.

Our results showed that PEE treatment cause increased PI31 gene expression, suggesting that proteasome activity might be inhibited in PEE-treated UBUC cell. Suppression of proteasome activity may prevent activation/translocation of NFκB to the nucleus to activate downstream pathways [18]. Results of western immunoblotting against RelA/p65 component of NF-κB demonstrated that PEE treatment for 48 h decreased the amount of RelA/p65 both in the cytoplasm and nucleus of T24 cell (Fig. 4). RelA/p65 along with p50 subunit moves to nucleus to activate the target genes when NF-κB is activated.

In the apoptotic pathway induced by damaged mitochondria, mitochondria release multiple pro-apoptotic proteins, such as Smac/Diablo, AIF, HtrA2 and EndoG along with cytochrome C. Smac/Diablo can bind to X-linked inhibitor of apoptosis protein (XIAP) and thus prevent XIAP to inhibit apoptosis [19]. Our results demonstrated that PEE incubation up-regulated Smac/Diablo expression (Fig. 3) while reduced XIAP expression (Fig. 4) in T24 cell and Diablo amount was also increased in PEE-treated TSGH8301 cell (Fig. 5), implicating that PEE treatment could induce apoptosis through damaged mitochondria.

## Discussion

Our previous studies have shown that PEE treatment could inhibit UBUC via evoking cell apoptosis [12]. In this study, we further found that PEE treatment could restrict UBUC cell proliferation and migration. To investigate the specific proteins affected by PEE incubation in UBUC cells, gel-based proteomics was carried out to shed light on the molecular mechanism underlying cancer intervention by PEE exposure through provoking cell apoptosis and inhibiting cell proliferation/migration. In this research, 20 differentially expressed proteins were found upon treatment of PEE to T24 cells with 19 up-regulated and 1 down-regulated proteins respectively.

Among de-regulated proteins, PHPT1 protein plays a role in lung cancer cell migration/invasion and is revealed to be associated with cytoskeleton reorganization [20]. Profilin 1 and Cofilin are the members of a family of actin-binding proteins, which participate in dynamic turnover and restructuring of actin cytoskeleton [15, 21]. Transgelin-2 is a cytoskeletal protein with actin-binding activity shown to be a tumor suppressor in colorectal carcinoma [22]. It has been postulated that cytoskeleton remodeling plays a pivotal role in cancer cell migration and also in regulating the morphologic and phenotypic events of a malignant cell. Besides, apoptosis is generally preceded by the pronounced changes of actin cytoskeleton [23]. Consistent with the above observations, results of this examination suggested that PEE treatment might provoke the rearrangement of cytoskeleton structure of UBUC cells through disturbing the expressions of cytoskeletal components and thus retard UBUC cell proliferation/migration. This study provided a clue for more investigation of the impacts of PEE on cytoskeleton structure in UBUC cell.

Previous documented findings demonstrated that augmented profilin 1 synthesis can increase PTEN gene expression in breast cancer cell [15]. PTEN protein serves as a phosphatase to dephosphorylate PIP_3_ to become PIP_2_. This dephosphorylation results in inhibition of Akt protein activity and thus Akt signaling pathway which plays a central role in protein synthesis, metabolism and cell proliferation [16]. Akt can phosphorylate tuberin/TSC2 to prevent the inhibition of mTORC1 complex (mTOR-raptor complex). mTORC1 integrates multiple signals to promote either cellular growth in favorable conditions or catabolic processes in unfavorable conditions while mTORC2 (mTOR-rictor complex) is related to actin organization. Many documented evidences indicated that impaired PTEN/Akt/mTOR signaling pathway plays a key role in tumorigenesis in many tumors [24]. In accordance with the aforementioned findings, our present data suggested that PEE treatment increased profilin 1 expression to up-regulate PTEN gene expression, which might in turn inhibited Akt/mTORC1 signaling pathway to prevent UBUC cell proliferation/migration.

The abnormal proteasomal activity contributes to tumorigenesis by offering cancer cell with anti-apoptotic protection and a survival advantage [25]. Our findings implicated that PEE treatment could alter the expressions of several genes associated with 26S proteasome activity (PSMD9, UBQLN1, PSMF1) in UBUC cells to disturb cell proliferation. PSMF1/PI31 can bind to 20S catalytic particle of 26S proteasome to hinder substrate access to the enzymatic core and thus results in the inhibition of proteasomal activity [26]. PSMD9 is a proteasomal assembly chaperone [27]. Ubiquilin-1 is thought to functionally link the ubiquitination machinery to the proteasome to effect in vivo protein degradation [28]. Suppression of proteasomal activity may prevent degradation of IκB (endogenous inhibitor of NF-κB) and subsequent activation/translocation of NFκB to the nucleus to activate downstream pathways [29]. NFκB plays an important role in tumorigenesis of many tumors by promoting cell proliferation, migration and suppression of apoptosis [18]. Our study indicated that PEE treatment could inhibit NFκB activation possibly through evoking PSMF1/PI31 over-expression to prevent the proteasomal degradation of IκB.

Among PEE-induced de-regulated proteins, BCL10, diablo, peflin, TPD52L2, eIF5A and BAG2 are shown to be involved in regulating cell apoptosis. Several findings suggested that BCL10 is an apoptotic regulatory protein and participates in Apaf1/caspase 9-mediated cell death pathway [30]. eIF5A is the only known protein to be regulated by the post-translational formation of a hypusine residue. Recent studies have indicated that unhypusinated eIF5A is pro-apoptotic and only observed during apoptosis [31]. Our proteomics data showed that PEE incubation could increase eIF5A gene expression but further investigation was required to determine whether unhypusinated eIF5A level was increased in PEE-treated T24 cell. TPD52L2 can interact with TPD52L1 protein which positively regulates apoptosis signal-regulating kinase 1 (ASK1)-induced apoptosis [32]. Peflin can regulate the activity of apoptosis-linked gene 2 (ALG-2) which associates with Fas-executed apoptosis [33]. In the apoptotic pathway, the caspapse activities can be inhibited directly by inhibitor of apoptosis (IAP) protein [19]. During the mitochondrial apoptotic process, the inhibitory function of XIAP, a ubiquitous member of IAP family, can be antagonized by Smac/Diablo and Omi/HtrA2 which are also released from mitochondria along with cytochrome c. BAG2 exhibits pro-apoptotic properties and is demonstrated to be up-regulated in proteasome inhibitor-induced apoptosis in thyroid carcinoma cell [34]. Our present results demonstrated that PEE exposure could increase the aforementioned apoptotic proteins to provoke UBUC cell apoptosis.

Some of dys-regulated proteins evoked by PEE treatment might attribute to the mitochondrial damage and nuclear change (nuclear fragmentation, chromatin condensation and DNA fragmentation) which are the characteristics of cell apoptosis. Translin/TRAX protein complex (C3PO) plays roles in very important key cellular processes such as cell growth regulation, genome stability regulation and carcinogenesis [35]. NAUFAF1 is a chaperone protein involved in the assembly of the mitochondrial NADH:ubiquinone oxidoreductase complex (complex I) which transfers the electron from NADH to ubiquinone (coenzyme Q) in the first step of the mitochondrial respiratory chain [36]. Cancer cells proliferate very rapidly and rely on high metabolic activities. To meet high energy demand, tumor cells exploit aerobic glycolysis to acquire the energy from glucose (Warburg effect). During glycolysis only one of two triosephosphates formed by aldolase-glyceraldehyde-3-phosphate-is degraded in the subsequent steps. The other product, dihydroxyacetone phosphate, is rapidly and reversibly converted to glyceraldehyde-3-phosphate by TPI1 protein [37].

## Conclusion

In conclusion, this study showed that exposure of UBUC cell to PEE might result in (1) apoptosis; (2) proteasome structure/activity alteration; (3) cytoskeleton rearrangement; (4) AKT/mTOR signaling pathway inhibition and (5) impaired aerobic glycolysis. Besides, IPA analyses showed that the de-regulated proteins were involved in apoptosis, proliferation, glycolysis, DNA metabolism. The results of this study provide a global picture to further investigate the anticancer molecular mechanism of PEE.

## Additional files

Additional file 1: This file included extended experimental procedures as well as materials and additional 2-DE gel pairs. (DOCX 276 kb) Additional file 2: Table S1. Statistical data for de-regulated proteins. **Table S2**. Detail mass spectrometry data of matched peptides for protein identification. (DOCX 28 kb)

## Abbreviations

ALG-2: apoptosis-linked gene 2
Akt/PKB: protein kinase B
BAG2: BCL2-associated athanogene 2
BCL 10: B-cell lymphoma/leukemia 10
CAPZA1: F-actin-capping protein subunit alpha-1
CFL1: C ofilin
DUT: dUTP pyrophosphatase
eIF5A: eukaryotic translation initiation factor 5A
HIF-1α: hpoxia-inducible factor 1-α
IEF: isoelectric focusing
IAP: inhibitor of apoptosis
LC-MS/MS: liquid chromatography-tandem mass spectrometry
mTORC1: mammalian target of rapamycin complex 1
NDUFAF1: NADH dehydrogenase (ubiquinone) 1 α subcomplex assembly factor 1
PE: pomegranate polyphenols, ellagitannin-rich extract
PEE: pomegranate fruit ethanol extract
PEF1: peflin
PFDN5: prefoldin subunit 5
PFE: Ellagitannin-rich Pomegranate fruit extract
PFN1: profilin 1
PHPT1: phosphohistidine phosphatase 1
POLE3: DNA polymerase ε subunit 3
PSMD9: 26S proteasome non-ATPase regulatory subunit 9
PSA: postate-specific antigen
PSMF1/PI31: proteasome inhibitor subunit 1
PTEN: phosphatase and tensin homolog
TAGLN2: transgelin-2
TPD52L2: tumor protein D54
TPI1: triose phosphate isomerase 1
TSNAX: translin-associated protein X
UBUC: urinary bladder urothelial carcinoma
UBQLN: ubiquilin 1
VEGF: vascular endothelial growth factor
XIAP: X-linked inhibitor of apoptosis protein
