# Supplementary material for: Clarification of the molecular pathway of Taiwan local pomegranate fruit juice underlying the inhibition of urinary bladder urothelial carcinoma cell by proteomics strategy
Source: BMC Complement Altern Med. 2016 Mar 9;16:96. doi: 10.1186/s12906-016-1071-7 (PMC4784391; doi:10.1186/s12906-016-1071-7)
Supplement: Additional file 1: — This file included extended experimental procedures as well as materials and additional 2-DE gel pairs. (DOCX 276 kb) [file 12906_2016_1071_MOESM1_ESM.docx]

**Extended experimental procedures and materials**

**Cell counting assay**

A 6-well plate was seeded with 8 × 10^4^ T24 or J82 cells per well. After cultivation for the indicated time period, cell number was counted with BeckmanCoulter Z1 Counter (BeckmanCoulter Inc., Indianapolis, IN, USA). Three wells were exploited for a specific time period.

**3-(4,5-**[**di**](http://en.wikipedia.org/wiki/Di-)[**methyl**](http://en.wikipedia.org/wiki/Methyl)[**thiazol**](http://en.wikipedia.org/wiki/Thiazole)**-2-yl)-2,5-di**[**phenyl**](http://en.wikipedia.org/wiki/Phenyl)**tetrazolium bromide (MTT) assay**

Appropriate concentrations of PEE were added to a 96-well plate already seeded with 5,000 human T24 cells or 6,000 human J82 cells per well. After exposure for the indicated time duration, 20 μl of MTT solution (Merck, Damstadt, German) (5 mg/ml PBS) was added to each well and the plate was incubated at 37ºC for 4 hours. After medium removal, 200 μl of DMSO was added to each well and the plate was gently shaken for 5 minutes. The absorbance was determined at 540 nm. Quadruplicate wells were applied to each concentration for a specific time period. 0.5 % (v/v) DMSO (vehicle)-treated bladder cancer cells were employed as the control.

**Wound scratch assay**

2.1×10^4^ 0.5 % (v/v) DMSO- or 2.1×10^4^ 50 μg/mL PEE-treated human T24 cells were plated onto each well with culture-insert (Ibidi, Germany) of a 6-well plate and cultivated overnight in McCoy's 5A medium containing 1 % (v/v) FBS. The culture-insert was removed by sterile tweezers and 2 ml fresh medium was added to each well. We measured the distances of the initial gaps at 0-hour and the final gaps at 10-hour under a phase contrast microscope (Nikon, Tokyo, Japan). The relative cell migration capability was transformed to absolute migration capability. Absolute migration capability (μm/h)＝(G_0_－G_t_)/2t, where G_0_ is the initial gap (μm) at 0-hour and G_t_ is the final gap for a time period of 10 hours. Experiments were performed in triplicate.

**LavaPurple staining**

The LavaPurple staining was performed according to manufacture’s suggested protocol (Fluorotechnics, Sydeny, Australia). Briefly after 2-DE, the gels were incubated with solution I [(1 % (w/v) citric acid and 15 % (v/v) ethanol) for 1.5 hours and then soaked in solution II [0.63 % (w/v) boric acid, 0.385 % (w/v) and 0.5 % (v/v) Lavapurple] for 1.5 hours under dark. Lastly the gels were incubated in solution III [15 % (v/v) ethanol] for 30 minutes and then in solution I for more 30 minutes.

**Preparation of protein lysates for two-dimensional gel electrophoresis**

70%-80% confluent T24 cells were incubated with 50 μg/mL PEE for 36 hours. T24 cells were then collected by centrifugation at 1,000 ×g for 5 minutes at 25°C and lysed with lysis solution [7 M urea, 2 M thiourea, 100 mM dithiothreitol (DTT), 4 % (v/v) CHPAS, 40 mM Tris-base (pH 10), 1 mM PMSF, and 1 Complete Mini protease inhibitor cocktail tablet (Roche, Diagnostics, Indianapolis, USA) per liter] with shaking at room temperature for 1 hour. Then the lysates are ultracentrifuged at 349,000 ×g for 2 hours at 15 °C in Type 90 Ti rotor (Beckman Coulter, Fullerton,CA, USA) and the supernatant was precipitated with 2-D Clean-up Kit (GE Healthcare Bio-Sciences AB, Uppsala, Sweden) according to the manufacture’s protocol. Protein amount in the resulting protein lysate was measured by Bio-Rad DC protein assay. The protein lysates were kept at -80°C until use. 0.5 % (v/v) DMSO (vehicle)-treated T24 cells were implemented as the control.

**Preparation of nuclear and cytoplasmic extracts**

Nuclear and cytoplasmic protein extracts were isolated from DMSO-treated or PEE-treated T24 cells using Nuclear/Cytosol Fractionation Kit (BioVision, Mountain view, CA, USA) according to the protocol suggested by the manufacture.

**Commercial Antibodies**

Diablo (1:1000) was from Enzo, Farmingdale, NY, USA. Profilin 1 (1:1000), NDUFAF1 (1:10000) and PTEN (1:1000) were from Epitomics, Burlingame, California, USA. Triosephosphate isomerase (1:200) was from Santa Cruz, Dallas, Texas, USA. PSMF1 (1:200) was from Santa Cruz, Dallas, Texas, USA. P-Akt (S473, 1:2000), actin (1:5000) and mTOR P-S2448 (1:1000) were from Millipore, Billerica, MA, USA. Akt (1:1000) and XIAP (1:500) were from GeneTex, Irvine, CA, USA.JNK (1:1000) was from BD Biosciences, San Jose, CA, USA.P-JNK(1:1000) was from Cell signaling, Danvers, MA, USA.


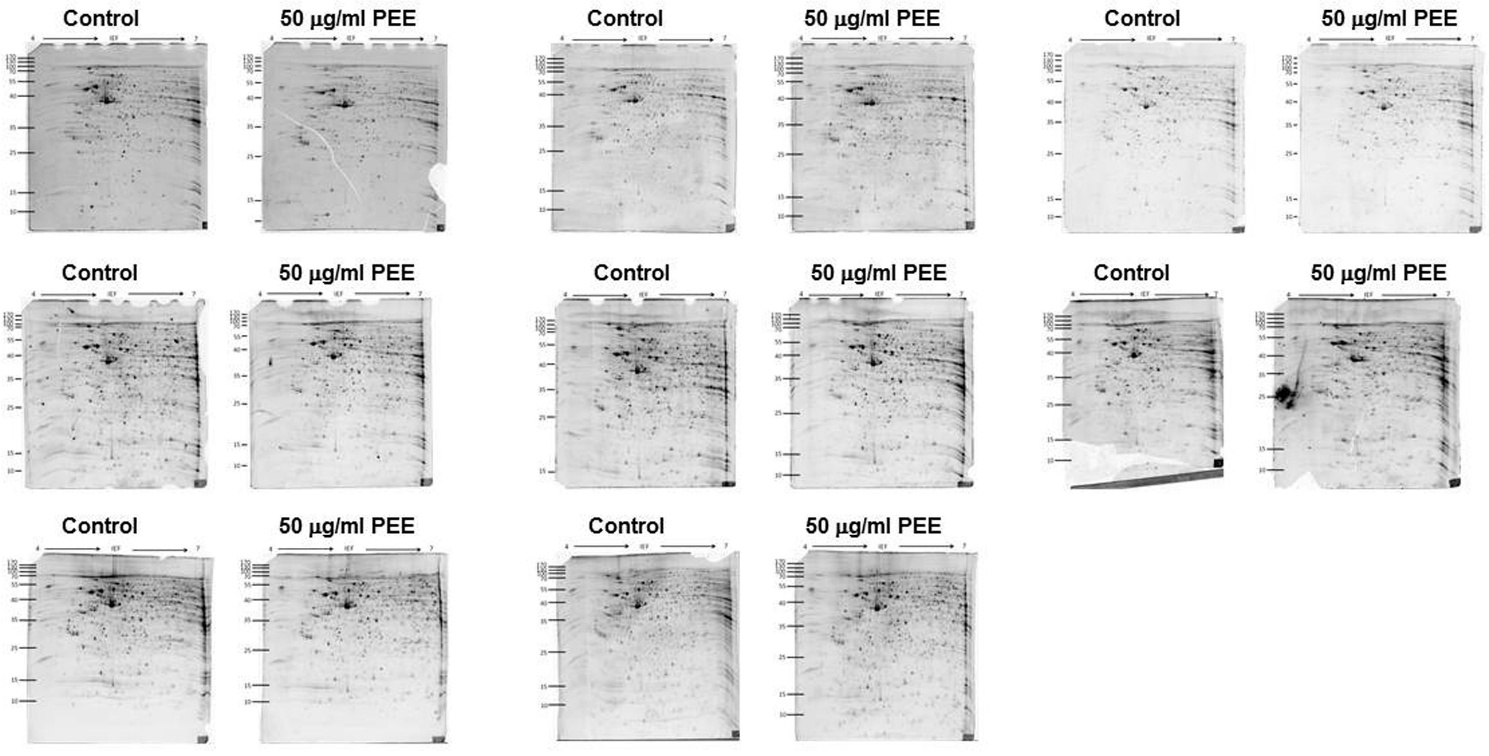


**Figure S1. Additional 2-DE pairs.**
