# Supplementary material for: Clarification of the molecular pathway of Taiwan local pomegranate fruit juice underlying the inhibition of urinary bladder urothelial carcinoma cell by proteomics strategy
Source: BMC Complement Altern Med. 2016 Mar 9;16:96. doi: 10.1186/s12906-016-1071-7 (PMC4784391; doi:10.1186/s12906-016-1071-7)
Supplement: Additional file 2: Table S1. — Statistical data for de-regulated proteins. Table S2. Detail mass spectrometry data of matched peptides for protein identification. (DOCX 28 kb) [file 12906_2016_1071_MOESM2_ESM.docx]

| **Spot** | **Incidences** | **Control**  **volume (ppm)**  **(mean ± S.D.)^a^** | **PEE**  **volume (ppm)**  **(mean ± S.D.)^a^** | ***P-*value** | **Fold** |
| --- | --- | --- | --- | --- | --- |
| 1 | 5/9 | 12.14±4.06 | 33.14±10.81 | 0.0036 | +2.73 |
| 2 | 5/9 | 8.82±11.85 | 66.88±32.45 | 0.0056 | +7.58 |
| 3 | 6/9 | 1.26±0.77 | 2.25±0.63 | 0.036 | +1.78 |
| 4 | 6/9 | 5.57±3.65 | 71.48±56.97 | 0.081 | +12.84 |
| 5 | 6/9 | 33.23±36.83 | 170.1±97.17 | 0.018 | +5.12 |
| 6 | 5/9 | 23.52±17.28 | 93.34±34.04 | 0.0091 | +3.97 |
| 7 | 5/9 | 28.16±21.33 | 81.62±34.55 | 0.0035 | +2.9 |
| 8 | 5/9 | 10.5±11.46 | 71.48±25.07 | 0.019 | +6.81 |
| 9 | 5/9 | 0.840.77 | 2.10±0.47 | 0.0011 | +2.51 |
| 10 | 7/9 | 10.5±7.45 | 66.92±47.15 | 0.014 | +6.31 |
| 11 | 5/9 | 31.37±37.73 | 196.57±149.01 | 0.03 | +6.27 |
| 12 | 5/9 | 5.26±4.43 | 37.64±16.79 | 0.021 | +7.17 |
| 13 | 5/9 | 19.24±22.68 | 100.04±69.44 | 0.039 | +5.2 |
| 14 | 5/9 | 78.6±32.20 | 251.06±60.38 | 0.0005 | +3.2 |
| 15 | 5/9 | 29.12±21.88 | 114.7±70.44 | 0.032 | +3.94 |
| 16 | 5/9 | 16.26±12.18 | 76.64±47.61 | 0.025 | +4.71 |
| 17 | 5/9 | 9.34±7.11 | 61.06±29.35 | 0.005 | +6.54 |
| 18 | 5/9 | 1.6±0.6 | 2.52±0.46 | 0.027 | +1.57 |
| 19 | 5/9 | 559.26±236.09 | 1601.12±572.72 | 0.0055 | +2.86 |
| A | 5/9 | 1.71±0.41 | 0.88±0.67 | 0.046 | –1.93 |

**Table S1.** Differentially expressed proteins in PEE-treated T24 cells

^a^ S.D. : standard deviation. The total normalized volume was evaluated by the intensity of each spot divided by the sum of intensities of all spots in the gel.

**Table S2.**The detail mass spectrometry data of matched peptides

| Spot | Protein I.D. | Matched^b^ peptide number | Sequence^a^ |
| --- | --- | --- | --- |
| 1 | ubiquilin 1 (UBQLN1) | 10 | EANLQALIATGGDINAAIER ( 2,6.42,0.68,1)  FQQQLEQLSAMGFLNR (2,6.37,0.57,1)  ALSNLESIPGGYNALR (2,5.61,0.57,1)  NPEISHMLNNPDIMR (2,5.23,0.59,1)  QQLPTFLQQMQNPDTLSAMSNPR (2,5.20,0.61,1)  QLIMANPQMQQLIQR (2,4.98,0.55,1)  SHTDQLVLIFAGK (2,4.41,0.53,1)  EKEEFAVPENSSVQQFK (2,3.46,0.54,1)  NPAMMQEMMR (2,3.38,0.59,1)  QTLELAR (2,1.17,0.09,27) |
| 2 | DNA polymerase ε subunit 3 (POLE3) | 6 | TLNASDVLSAMEEMEFQR (2,4.8,0.65,1)  IIKEALPDGVNISK (2,4.52,0.51,1)  IIKEALPDGVNISKEAR (2,3.27,0.43,1)  EALPDGVNISKEAR (2,2.53,0.49,1)  EALPDGVNISK (2,2.30,0.38,2)  FVTPLKEALEAYR (2,2.18,0.24,1) |
| 3 | eukaryotic translation initiation factor 5A (eIF5A) | 4 | NDFQLIGIQDGYLSLLQDSGEVR (2,5.39,0.65,1)  VHLVGIDIFTGK (2,3.14,0.60,1)  EDLRLPEGDLGK (2,2.46,0.29,1)  IVEMSTSK (2,2.01,0.25,4) |
| 4 | tumor protein D54 (TPD52L2) | 8 | KTQETLSQAGQK (2,4.19,0.51,1)  TSAALSTVGSAISR (2,4.16,0.52,1)  SWHDVQVSSAYVK (2,4.00,0.50,1)  TQETLSQAGQK (2,3.61,0.41,1)  VVGDRENGSDNLPSSAGSGDKPLSDPAPF (3,3.58,0.30,1)  LGLSTLGELK (2,3.13,0.40,1)  QVLAAKER (2,2.20,0.11,84)  KLGDMR (2,1.90,0.26,5) |
| 5 | phosphohistidine phosphatase 1 (PHPT1) | 2 | AKYPDYEVTWANDGY (2,4.56,0.54,1)  SGAPAAESKEIVR (2,3.54,0.48,1) |
| 6 | proteasome inhibitor subunit 1 (PSMF1/PI31) | 6 | IVSGIITPIHEQWEK (2,4.82,0.11,1)  ALIDPSSGLPNRLPPGAVPPGAR (3,4.70,0.49,1)  TYKNSEELR (2,3.24,0.25,1)  ANVSSPHR (2,2.23,0.26,1)  RGGMIVDPLR (2,2.15,0.10,48)  GGMIVDPLRSGFPR (2,1.47,0.03,116) |
| 7 | B-cell lymphoma/leukemia 10 (BCL 10) | 3 | SNSDESNFSEKLR (2,3.66,0.49,1)  TQNFLIQK (2,2.53,0.26,1)  ITDEVLK (2,1.91,0.13,12) |
| 8 | dUTPpyrophosphatase (DUT) | 5 | IFYPEIEEVQALDDTER (2,5.8,0.58,1)  ARPAEVGGMQLR (3,4.39,0.49,1)  AAGYDLYSAYDYTIPPMEK (2,4.34,0.63,1)  LSEHATAPTR (3,3.29,0.44,1)  IFYPEIEEVQALDDTERGSGGFGSTGKN (3,2.99,0.51,1) |
| 9 | prefoldin subunit 5 (PFDN5) | 9 | NQLDQEVEFLSTSIAQLK (2,6.96,0.66,1)  IQQLTALGAAQATAK (2,5.95,0.59,1)  ELLVPLTSSMYVPGK (2,3.43,0.66,1)  QMEKIQPALQEK (2,3.10,0.11,2)  QAVMEMMSQK (2,2.53,0.52,1)  TAEDAKDFFKR (2,2.37,0.23,29)  KIDFLTK (2,2.23,0.20,8)  IDFLTK (2,1.68,0.13,2)  IQPALQEK (2,1.01,0.08,121) |
| 10 | peflin (PEF1) | 7 | LSFEDFVTMTASR (2,4.94,0.65,1)  IDVYGFSALWK (2,4.51,0.49,1)  SANPAMQLDR (2,3.03,0.40,1)  EKDTAVQGNIR (2,2.90,0.40,1)  DTAVQGNIR (2,2.64,0.28,1)  NLFQQYDR (2,2.60,0.30,1)  FIQQWK (2,2.20,0.03,41) |
| 11 | triosephosphate isomerase 1 (TPI1) | 8 | HVFGESDELIGQK (2,4.32,0.63,1)  TATPQQAQEVHEKLR (2,4.32,0.41,1)  VPADTEVVCAPPTAYIDFAR (3,4.06,0.53,1)  TATPQQAQEVHEK (2,3.61,0.38,1)  VVLAYEPVWAIGTGK (2,3.59,0.56,1)  LDEREAGITEK (3,3.40,0.41,1)  VIADNVKDWSK (2,3.24,0.37,1)  ELASQPDVDGFLVGGASLKPEFVDIINAK (3,1.91,0.17,2) |
| 12 | 26S proteasome non-ATPase regulatory subunit 9 (PSMD9) | 2 | SDVDLYQVR (2,3.29,0.46,1)  KLGQSESQGPPR (2,3.00,0.36,1) |
| 13 | NADH dehydrogenase (ubiquinone) 1 αsubcomplex assembly factor 1 (NDUFAF1) | 4 | NNQSALLYGTLSSEAPQDGESTR (2,5.85,0.73,1)  EVALDITSSEEKPDVSFDK (2,5.58,0.62,1)  GGPYWQEVK (2,2.55,0.08,11)  VVWQFR (2,1.51,0.39,1) |
| 14 | transgelin 2 (TAGLN2) | 10 | YGINTTDIFQTVDLWEGK (2,6.43,0.62,1)  NFSDNQLQEGKNVIGLQMGTNR (2,4.28,0.54,1)  DDGLFSGDPNWFPK (2,4.27,0.56,1)  GASQAGMTGYGMPR (2,4.10,0.56,1)  TLMNLGGLAVAR (2,3.98,0.45,1)  NFSDNQLQEGK (2,3.69,0.41,1)  GPAYGLSR (2,2.29,0.49,1)  KDVGRPQPGR (3,1.94,0.19,36)  DVGRPQPGR (2,1.92,0.09,62)  EVQQKIEK (2,1.87,0.15,8) |
| 15 | Diablo (DIABLO) | 10 | MNSEEEDEVWQVIIGAR (2,5.36,0.5,1)  LQVEEVHQLSR (2,4.33,0.52,1)  LAEAQIEELRQK (2,4.15,0.27,1)  SEPHSLSSEALMR (2,3.94,0.51,1)  TQEEGEERAESEQEAYLRED (2,3.75,0.321,1)  AVYTLTSLYR (2,3.48,0.53,1)  AESEQEAYLRED (2,3.32,0.27,1)  HQEYLK (2,2.19,0.22,1)  NHIQLVK (2,1.95,0.22,4)  QYTSLLGK (2,1.03,0.03,129) |
| 16 | BCL2-associated athanogene 2 (BAG2) | 9 | IIDEVVNKFLDDLGNAK (2,5.16,0.5,1)  LLESLDQLELR (2,3.82,0.43,1)  TLQQNAESRFN (2,3.32,0.51,1)  RLETLLR (2,2.78,0.09,2)  EAATAVEQEK (2,2.68,0.42,1)  NPQQQESLK (2,2.48,0.34,1)  GAGSKTLQQNAESR (2,2.43,0.39,1)  QISDGEREELNLTANR (3,2.21,0.24,5)  LLEHSK (2,1.56,0.08,1) |
| 17 | translin-associated factor X (TSNAX) | 8 | VTPVDYLLGVADLTGELMR (2,6.51,0.65,1)  QVYDGFSFIGNTGPYEVSK (2,5.40,0.67,1)  AITTGLQEYVEAVSFQHFIK (2,4.74,0.60,1)  TPSSDAQDKQFGTWR (2,4.72,0.58,1)  ITSAPDMEDILTESEIKLDGVR (2,4.23,0.55,1)  DVNSSSPVMLAFK (2,4.15,0.61,1)  TIFLLHR (2,2.41,0.30,2)  DITVESKR (2,2.05,0.32,5) |
| 18 | profilin 1 (PFN1) | 7 | TFVNITPAEVGVLVGKDR (2,5.88,0.52,1)  DSLLQDGEFSMDLR (2,4.39,0.54,1)  STGGAPTFNVTVTK (2,3.3,0.56,1)  DSPSVWAAVPGK (2,3.28,0.35,1)  SSFYVNGLTLGGQK (2,2.99,0.25,1)  TLVLLMGK (2,2.26,0.40,2)  EGVHGGLINKK (2,2.04,0.13,44) |
| 19 | cofilin (CFL1) | 10 | EILVGDVGQTVDDPYATFVK (2,6.51,0.65,1)  NIILEEGKEILVGDVGQTVDDPYATFVK (3,6.10,0.64,1)  LGGSAVISLEGKPL (2,4.27,0.55,1)  YALYDATYETK (2,3.52,0.58,1)  KSSTPEEVKK (3,2.81,0.12,2)  SSTPEEVKKR (2,2.33,0.16,1)  EDLVFIFWAPESAPLK (2,2.25,0.06,217)  VFNDMK (2,1.85,0.18,5)  MIYASSK (2,1.70,0.22,1)  NIILEEGK (2,1.39,0.06,16) |
| A | F-actin-capping protein subunit alpha-1 (CAPZA1) | 10 | IIENAENEYQTAISENYQTMSDTTFK (2,5.67,0.73,1)  FITHAPPGEFNEVFNDVR (3,4.90,0.45,1)  KEASDPQPEEADGGLK (2,4.73,0.55,1)  EASDPQPEEADGGLK (2,4.00,0.53,1)  LLLNNDNLLR (2,3.28,0.24,1)  DVQDSLTVSNEAQTAK (2,2.92,0.41,1)  FTITPPTAQVVGVLK (2,2.85,0.49,1)  VSDEEKVR (2,2.54,0.23,6)  EASDPQPEEADGGLKSWR (3,1.45,0.10,1)  QLPVTR (2,1.00,0.13,11) |

^a^ the numbers in parentheses correspond to z, Xcorr, dCn, Rsp parameters of TurboSequest respectively. ^b^Matched peptide number: number of peptides matched with protein in MS/MS query.
